# Efficacy and safety of a modified ozone-mediated neurolysis in treatment for patients with resistant hypertension

**DOI:** 10.1038/s41440-025-02458-8

**Published:** 2025-11-14

**Authors:** Lishuai Zhang, Bin Huang, Yizhi Zhang, Fang Zeng, PIngge Tian, Biao Li, Xiwei Chen, Jiashuang Wang, Yihui Huang, Li Li

**Affiliations:** 1 Guangzhou Red Cross Hospital, Jinan University, Guangzhou, China; 2 Shanghai RenJi Hospital, Shanghai Jiaotong University, Shanghai, China

**Keywords:** lumbar ganglia, Ozone, Neurolysis, Resistant hypertension, Digital hypertension

## Abstract

The sympathetic ganglion was the initial target of renal denervation (RDN) for treating resistant hypertension (RH). In this pilot Phase II single-arm, open-label clinical trial, we developed a modified neurolysis technique based on one of the traditional pain-relieving techniques, CT-guided ozone-mediated neurolysis, to treat RH. A total of 45 patients were enrolled and underwent the operation, which involved the injection of an oxygen-ozone gas mixture around the lumbar/renal ganglia under CT guidance. Following the procedure, the antihypertensive medication burden (AHMB) decreased from 4.2 pre-procedure to 3.5 at 3 months and 3.2 at 6 months post-procedure. Additionally, at 3 months post-procedure follow-up, there was a significant decrease in the average 24-h mean systolic blood pressure (SBP) by –6.8 ± 2.1 mmHg, morning SBP/diastolic blood pressure (DBP) by –9.2 ± 2.3/–4.7 ± 1.3 mmHg, and daytime SBP/DBP by –8.0 ± 2.4/–2.9 ± 1.5 mmHg. By 6 months post-procedure follow-up, compared with pre-operation values, we observed a significant decrease in the average 24-h SBP by –5.53 ± 0.76 mmHg, morning SBP by –5.6 ± 2.1 mmHg, and daytime SBP/DBP by –7.2 ± 0.96/–2.3 ± 0.6 mmHg. We did not find significant reductions in the 24-h mean DBP, morning DBP, and nighttime SBP/DBP at the 6-month follow-up. No adverse events were observed during or after the procedure. We concluded that CT-guided ozone-mediated neurolysis targeting the renal-related lumbar ganglia is a promising alternative for treating RH, with advantages including being minimally invasive, contrast-free, non-invasive to the renal artery, and cost-effective.

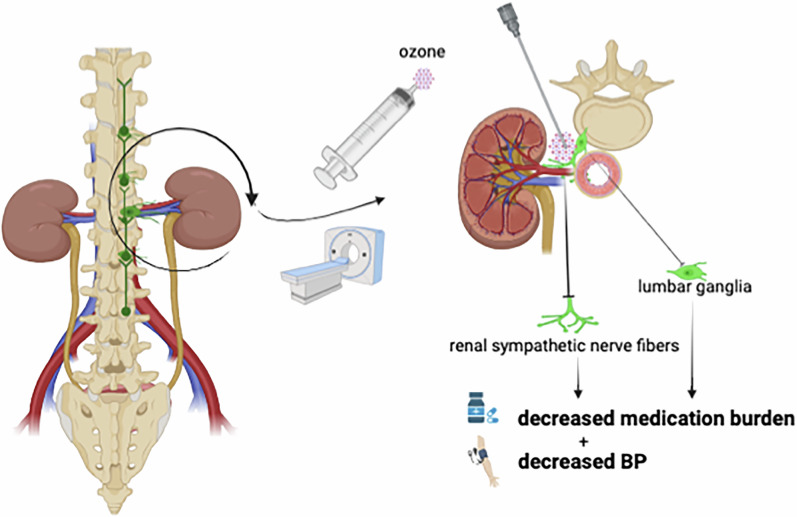

## Introduction

Elevated blood pressure (BP) stands as a pivotal risk factor for conditions such as ischemic heart disease, cerebrovascular disease, various cardiovascular disorders, progressive kidney dysfunction, and cognitive decline [1]. Resistant hypertension (RH) is defined as BP that remains above the target level despite the treatment with three or more antihypertensive medications at optimal doses, including a diuretic. It can also refer to cases where BP is controlled but requires four or more antihypertensive agents, indicating a high treatment burden [2]. As a result, RH imposes a substantial burden on both public health systems and individual patients.

Historically, the thoracic and lumbar sympathetic ganglia served as the primary anatomical targets for renal sympathetic denervation (RDN) in the treatment of RH [3]. Early surgical interventions, which involved sympathectomy through complete resection of the renal-related thoracic or lumbar sympathetic nerve chains and ganglia, have since been abandoned. In recent years, new minimally invasive or interventional techniques targeting the sympathetic ganglia have emerged to inhibit renal sympathetic activity for the treatment of hypertension and have demonstrated promising antihypertensive effects in preliminary studies [4, 5].

Ozone-mediated neurolysis has been widely used for pain relief and has gained recognition for its safety profile and efficiency, making it an attractive option in clinical practice [6, 7]. This technique involves the injection of an ozone-oxygen mixture around the sympathetic nerves. Notably, we observed that the anatomical site of this technique partially overlaps with the sympathetic nervous system innervating the kidneys, we modified the approach by focusing the injection site around the lumbar sympathetic ganglia associated with renal sympathetic innervation. Previously, in a single-arm clinical study, we observed that this modified technique, named CT-guided ozone-mediated lumbar-renal denervation (L-RDN), has the effect of BP lowering at 3-month follow-up and provided preliminary evidence of its safety in treating RH [8]. In the present study, we aim to further evaluate the 6-month antihypertensive efficacy and safety of this modified neurolysis technique.

## Methods

### Study design

This clinical trial is a prospective single-arm open-label clinical trial assessed the 6 months efficacy and safety of CT-guided ozone-mediated L-RDN in patients with RH. The inclusion criteria are as follows: (1) Age between 18 and 80 years. (2) Mean systolic BP (SBP) of ≥140 mmHg on 24-h ambulatory BP monitoring (ABPM) on at least three antihypertensive agents with maximally tolerated doses including a diuretic; or (3) Mean SBP ≤ 130 mmHg but on ≥4 antihypertensive agents. The exclusion criteria mainly include the following: (1) Secondary hypertension, such as renal artery stenosis, obstructive sleep apnea syndrome (OSAS), etc. (2) Severe cardiovascular and cerebrovascular diseases. (3) Spinal deformities or infections that cannot tolerate surgery. (4) history of renal artery angioplasty or denervation. (5) history of lumbar internal vertebral disc frequency ablation and sympathetic blockage. After the L-RDN, the criteria of reducing antihypertensive medication as following: the family SBP is ≤110 mmHg for two consecutive measurements during the follow-up. The study was approved by the ethics committee of Guangzhou Red Cross Hospital (No. 2022-145-01) and obtained the written informed consent from the patients.

### Adherence and the burden of antihypertensive medications (AHMB)

To ensure accurate statistical analysis of AHMB based on good adherence, all patients received standardized training on home BP measurement before discharge. They were instructed to measure and record their BP four times a day (upon waking up in the morning, at 10 am, at 4 pm, and before bedtime) using a standardized home BP log. Patients were required to strictly follow the discharge medication regimen and upload their home BP records weekly to a WeChat group that included both the follow-up doctor and the patient. Patients who failed to measure or upload their BP as required, or who independently altered the type or dosage of their antihypertensive medications, were excluded from the analysis. When the criteria for reducing medication are met, the reduction sequence, including the type and dose, is determined by the doctor based on individualized principles. Diuretics should be prioritized for retention, unless there are contraindications. The novel medication regimen is recorded and reported to the follow-up team. The total antihypertensive medication, expressed in standard dosage, type (SDT), was summed at 3 months and 6 months post-operation. The average AHMB was calculated as the total cumulative SDT divided by the number of participants at each time point.

### L-RDN procedure

The modified CT-guided ozone-mediated neurolysis procedure follows the protocol described in our previous publication [9].

### Blood pressure measurements

Patients underwent ABPM (MC 6800. Shenzhen Mindray Biomedical Electronics Co., Ltd) prior to the procedure, with follow-up measurements performed at 3 and 6 months. The ABPM data are categorized and analyzed based on morning, daytime, and evening periods.

### Safety

All adverse events were systematically reported and documented through phone calls, WeChat, or during outpatient visits. Potential complication encompasses orthostatic hypotension, gastrointestinal dysfunction, dysuria, and male erectile dysfunction, among others. Renal function, including eGFR, serum creatinine, and 24 h urinary protein, was routinely evaluated at 3- and 6- month follow-up.

### Statistical analysis

Statistical analyses were carried out using R statistical software version 4.1.3 (R Foundation for Statistical Computing, Vienna, Austria). Blood pressure measurements obtained before and after the procedure were compared using paired Student’s *t* tests. A two-tailed *p*-value of <0.05 was considered statistically significant, with significance evaluated separately for each hemodynamic parameter.

## Results

### Study population

**Fig. 1:**
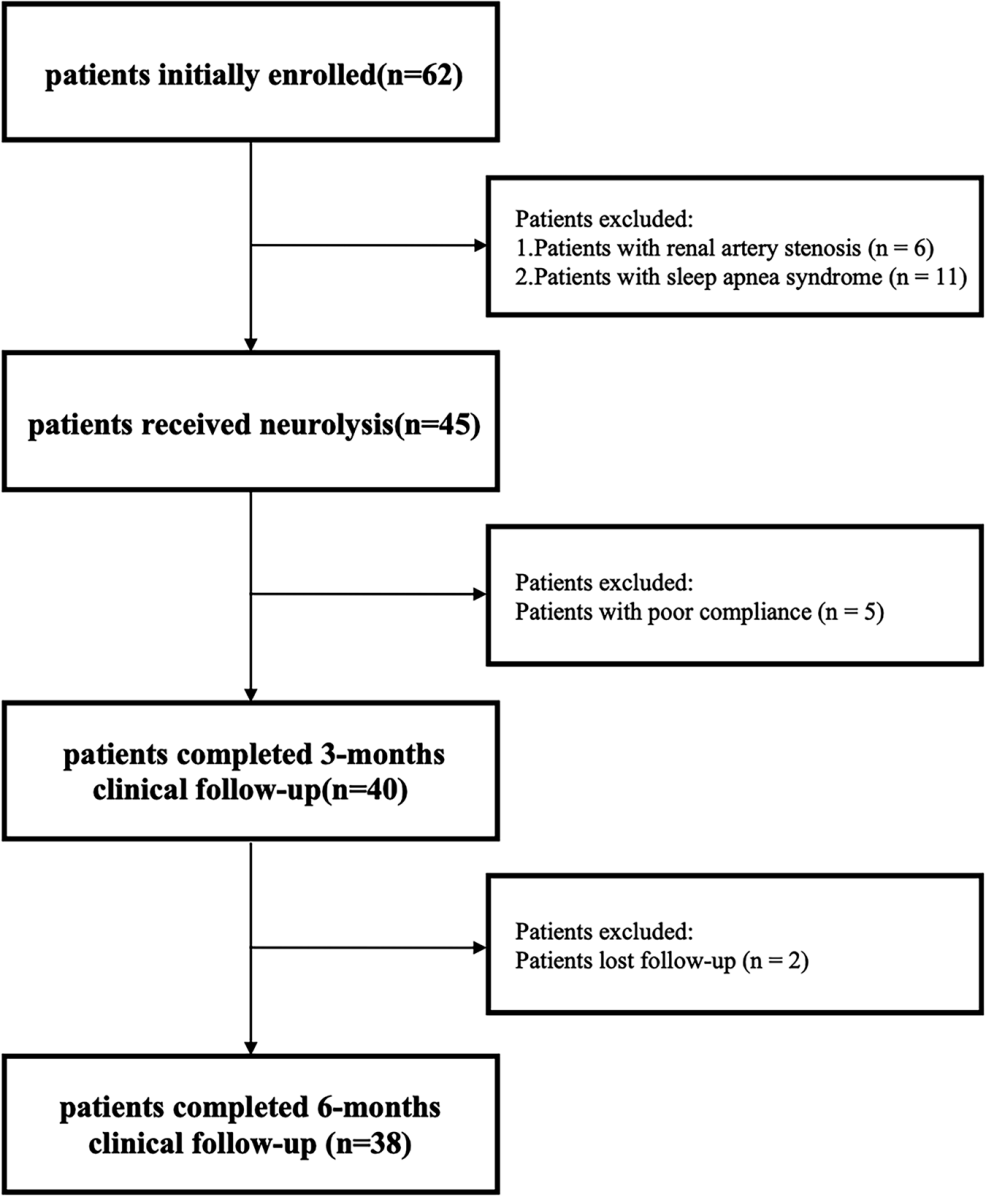
Initially, 62 patients were enrolled, with 6 cases of renal artery stenosis and 11 cases of OSAS excluded. A total of 45 patients underwent surgery. During the 3-month postoperative follow-up, 5 patients were excluded due to poor compliance. During the 6-month follow-up, 2 patients were lost to follow-up. Finally, 40 patients and 38 patients completed the 3-month and 6-month follow-ups, respectively

Of the 62 patients initially enrolled, 17 were excluded based on predefined criteria. including 6 with renal artery stenosis and 11 with OSAS. Forty-five patients were proceeded to receive the neurolysis procedure. Five patients were subsequently excluded due to poor adherence during the 3-month follow-up, leaving 40 patients who completed the 3-month follow-up. During the 3- to 6-month follow-up period, 2 additional patients were lost to follow up, resulting in 38 patients completing the 6 months follow-up. The patient enrollment flowchart is shown in Fig. 1.

### Baseline characteristics

Baseline characteristics of the participants are presented in Table 1. Of the cohort, 26 patients (57.8%) had uncontrolled hypertension (>140/90 mmHg) while on three SDT antihypertensive medications, and 19 patients (42.2%) had controlled hypertension (<140/90 mmHg) but were currently receiving at least four SDT antihypertensive medications. The maximum SDT taken by a single patient was seven. As a result, the baseline 24-h average BP across the cohort was below 140/90 mmHg.

**Table 1:** Baseline characteristics of the enrolled patients

Index	Enrolled patients (*n* = 45)
Age, years (SD)	64.00 (9.01)
Gender	
Male, *n* (%)	22 (48.89)
Female, *n* (%)	23 (51.11)
BMI, kg/m^3^ (SD)	25.88 (3.81)
Renal function	
eGFR < 60 mL/min/1.73 m^3^, *n* (%)	11 (24.44)
Dialysis, *n* (%)	2 (4.44)
Average eGFR (mL/min/1.73 m^3^)	74.39 (28.47)
T2DM, *n* (%)	17 (37.78)
ABPM Blood pressure, mmHg (SD)	
24 h mean SBP	133.27 (21.61)
24 h mean DBP	73.57 (12.17)
Antihypertensive medication burden, *n* (SD)	4.18 (1.37)
≥10 years history of HTN, *n* (%)	36 (80.00)

### Changes in the antihypertensive medication burden (AHMB)

The changes in total SDT and AHMB were summarized in Table 2. Among the study cohort, 75% of patients were taking four or more types of antihypertensive medications, with a maximum individual SDT and AHMB of 7.0. During the 3-month postoperative follow-up, 11 (27.5%) patients met the criteria for medication reduction, and an additional 6 patients achieved the criteria during the 3- to 6-month follow-up period. As showed in Table 2, the total SDT decreased from 210 preoperatively to 158 and 136 at 3- and 6- month follow-ups, respectively. Mean AHMB correspondingly decreased from 4.18 preoperatively to 3.5 and 3.25 at the end of 3- and 6- month follow-ups.

**Table 2:** Changes in total SDT and AHMB

Antihypertensive drugs	Pre-procedure (*n* = 45)	3 months follow-up (*n* = 40)	6 months Follow-up (*n* = 38)
CCB	63	42.5	36
ACEI/ARB	50	37.5	31.5
Diuretic	47	43	34.5
β-blockers	33.5	29.5	29.5
α-blockers and others	17	6	5
Total SDT	210.5	158.5	136.5*
AHMB	4.18	3.50	3.25

*SDT* standard dosage type

*P < 0.05

### Changes of BP in ABPM

**Fig. 2:**
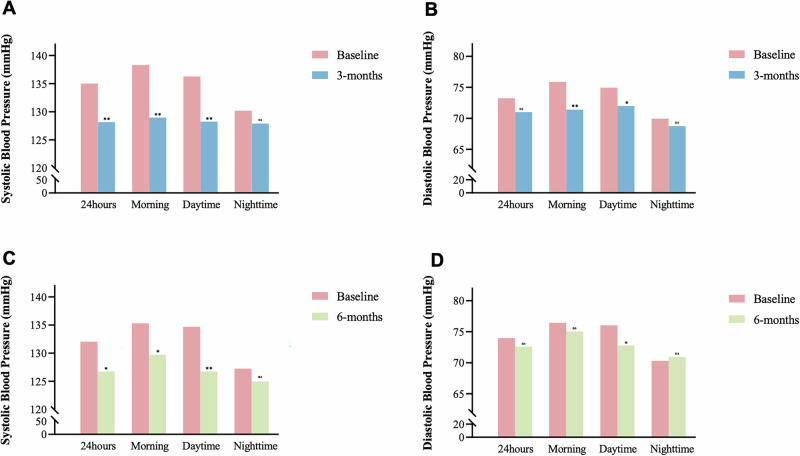
At 3-month follow-up, compared to pre-operation, SBP of 24 h mean, morning and daytime, DBP of morning and daytime decreased significantly reduced (**A, B**). At 6-month follow-up, compared to pre-operation, SBP of 24 h mean, morning and daytime, DBP of daytime remained significantly decrease (**C, D**). ***: compared with pre-operation: *p* < 0.001. **: compared with pre-operation: *p* < 0.01. *: compared with pre-operation: *p* < 0.05. ns *p* > 0.05

As illustrated in Fig. 2, at 3 months post-procedure, the average 24-h mean SBP decreased significantly (*Δ* = –6.8 ± 2.1 mmHg, *p* = 0.002), as did morning SBP/DBP (*Δ* = –9.2 ± 2.3/–4.7 ± 1.3 mmHg, *p* = 0.001/0.002) and daytime SBP/DBP (*Δ* = –8.0 ± 2.4/–2.9 ± 1.5 mmHg, *p* = 0.002/ = 0.047). Changes in 24 h mean DBP and nighttime SBP/DBP were observed but did not reach statistical significance (*p* > 0.05). At 6 months post-procedure, compared with baseline, significant reductions were observed in the average 24-h SBP (*Δ* = –5.53 ± 0.76/mmHg, *p* = 0.020), morning SBP/ (*Δ* = –5.6 ± 2.1, *p* = 0.023), and daytime SBP/DBP (*Δ *= –7.2 ± 0.96/–2.3 ± 0.6 mmHg, *p* = 0.002/0.010), whereas no significant changed were detected in 24-h mean DBP, morning DBP, or nighttime SBP/DBP (*p* > 0.05).

### Blood pressure reduction magnitude and corresponding MB

**Fig. 3:**
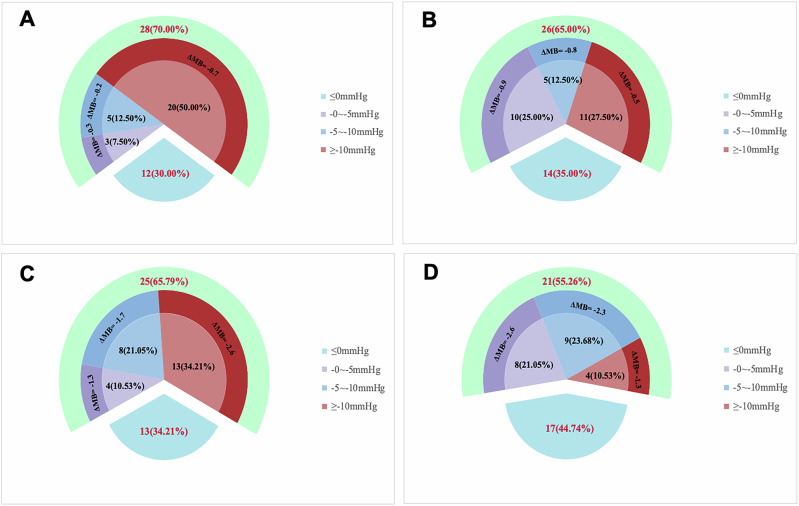
After 3 months, 70% and 65% of patients exhibited a reduction in 24-h mean systolic blood pressure (SBP) and diastolic blood pressure (DBP), respectively (**A, B**). Among them, 50% experienced a decrease in SBP greater than 10 mmHg, accompanied by a mean medication burden (MB) reduction of −0.7. Additionally, 27.5% showed a DBP reduction greater than 10 mmHg, with an associated MB decrease of −0.5. A further 12.5% of patients had reductions of 5–10 mmHg in both SBP and DBP, with corresponding MB decreases of −0.2 and −0.8, respectively. At six months post-procedure, 67% of patients demonstrated a reduction in 24-h mean SBP, and 55% showed a reduction in DBP (**C, D**). Specifically, 34% experienced an SBP decrease of more than 10 mmHg, along with a reduction in MB of −2.6. Additionally, 10% of patients had a DBP decrease greater than 10 mmHg, with a corresponding MB reduction of −1.3. Furthermore, 21.05% and 23.69% of patients achieved reductions of 5–10 mmHg in SBP and DBP, accompanied by MB decreases of −1.7 and −2.3, respectively

At three months, the majority of patients demonstrated reductions in 24-h mean SBP and DBP (70% and 65%, respectively), with nearly half achieving a SBP reductions of >10 mmHg. These improvements were accompanied by corresponding decreases in medication burden. Similar trends were observed at six months, with 67% and 55% of patients showing reductions in SBP and DBP, respectively. A substantial proportion of patients achieved BP reductions of >10 mmHg, which were associated with further decreases in medication use. The magnitude of BP reduction and the corresponding decrease in MB are illustrated in Fig. 3.

### Safety of L-RDN

**Fig. 4:**
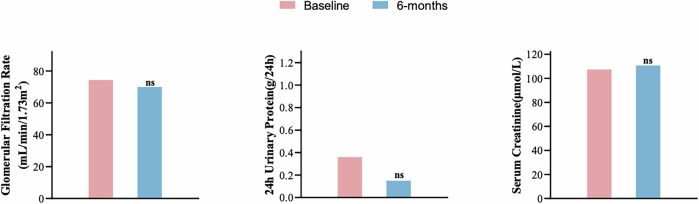
Compared to baseline value, eGFR, serum Cr and 24 h urinary protein did not change significantly after the procedure (p > 0.05)

During the procedure and throughout the 6-month follow-up period, no adverse events were observed. Additionally, there were no significant changes in eGFR, serum creatinine, or 24 h urine protein levels before and after the intervention. Renal function and urinary protein before and after the procedure are presented in Fig. 4.

## Discussion

The significant finding of this clinical trial is that the application of ozone to the lumbar sympathetic ganglia, which are associated with renal sympathetic innervation, led to a significant reduction in BP for patients with RH. This decrease in BP persisted from 3 to 6 months postoperatively. Additionally, neither our previous study nor this study has identified any significant adverse events associated with this technique.

The relationship between increased sympathetic nervous activity, particularly renal sympathetic activity, and hypertension has been well established [10]. Early surgical approaches aimed at lowering renal sympathetic activity to treat malignant hypertension involved extensive removal of the sympathetic nerve chains and ganglia innervating the kidneys [11]. Although these methods were eventually abandoned due to the invasive nature of the surgery and large extent of the excision, they nevertheless demonstrated the significant efficacy of RDN in treating hypertension. The currently popular catheter-based RDN technique via the renal arteries has been proven to effectively lower BP [12]. However, according to current guidelines, catheter-based RDN is not recommended for patients with an estimated glomerular filtration rate (eGFR) below 40 mL/min/1.73 m² because of the nature of depending contrast [13]. Additionally, its procedure is relatively complex and requires the use of specialized catheters, which contributes to higher costs. As a result, in recent years, RDN techniques targeting the sympathetic ganglia have regained attention [14, 15]. For instance, researchers have reported injecting alcohol into celiac ganglia of hypertensive mice—a neurolysis technique originally used for alleviating abdominal pain—produced significant antihypertensive effects [4].

CT-guided ozone-mediated neurolysis is a well-established conventional technique in orthopedics and pain management, and its safety in intradiscal or paraganglionic local injections has been confirmed through long-term clinical application [16]. Ozone, a potent oxidizing agent, exhibits anti-neuroinflammatory and anti-oxidative stress effects [17]. Recent studies indicate that high-concentration ozone promotes apoptosis of nerve cells [18]. In our previous study, we observed that the modified neurolysis of injecting ozone around lumbar ganglia at the level of the left renal artery effectively reduced 24-h ABPM values and improved home BP control at the 3-month postoperative follow-up [8, 9]. At the 6-month postoperative follow-up, one patient demonstrated a significant reduction in BP, accompanied by a decrease in antihypertensive medication burden from four drug categories (one of which was at double the standard dose) to two standard-dose medications [19]. Researchers have suggested that this modified neurolysis targeting the lumbar sympathetic ganglion may become a new treatment option for RH [20]. In this study, beyond targeting the lumbar sympathetic ganglia innervating the kidneys, adjacent anatomical structures include the aortorenal ganglia located at the intersection of the abdominal aorta and renal artery, as well as sympathetic nerve fibers distributed along the renal artery surface were also the potential targets of ozone application. In this study, the post-operative antihypertensive effect was rapid, with a more pronounced reduction in SBP compared to DBP. Additionally, we found that SBP decreased most significantly in the morning, and this effect was maintained up to 6 months after the procedure. These findings were similar to those observed in catheter-based RDN [21, 22].

In this study, we only injected ozone on the left side, yet it still demonstrated significant antihypertensive effects. This suggests that targeting the sympathetic nerves on one side of the kidney may be sufficient to achieve BP reduction. In contrast, for catheter-based RDN, unilateral denervation has been reported to be ineffective in lowering BP [23]. From an anatomical perspective, the abdominal aorta typically descends along the left side of the spine, which makes the left-side approach more accessible to the aortic renal ganglia and the adventitia of the renal arteries. Furthermore, the gaseous nature of ozone may facilitate diffuse contact with these structures, thereby enhancing its therapeutic effect.

The results of this study demonstrated that ozone-mediated neurolysis mimic the antihypertensive effects of RDN, although the underlying mechanism remains to be fully elucidated. Beyond reducing renal sympathetic activity, ozone may also exert RDN-like effects by modulating autonomic nervous function through anti-neuroinflammation or regulation of oxidative stress. This hypothesis is supported by recent evidence showing that neuroinflammation plays a pivotal role in the onset and progression of hypertension [24, 25]. Ongoing mechanistic studies and basic research are expected to provide further insights into these potential pathways.

Similar to other transarterial interventions, traditional RDN carries common catheter-related risks (e.g., access-site hematoma, arteriovenous fistula, aneurysm, dissection, or perforation) as well as some procedure-specific complications. In the Symplicity HTN-2 trial, RDN-related vascular complications included access-site injuries and two renal artery dissections [26]. While since this procedure is performed outside the renal artery and the access route does not involve the femoral artery, the potential risk of major vascular injury is effectively avoided. Specific incidence rates of contrast-induced nephropathy (CIN) are rarely reported in conventional RDN studies. Based on an average of 105 mL of contrast used per conventional RDN [27], the use of contrast agents remains a concern for patients with pre-existing renal impairment or those with high-risk factors for CIN. A new technique exploring contrast-free or low-contrast approaches to further prevent renal injury [28]. In contrast, this technique is performed under CT guidance instead of contrast, thus avoiding the risk of CIN and being applicable even to patients with impaired renal function, including those with severe renal dysfunction, even on dialysis. Moreover, it does not involve specialized catheters but only requires a standard puncture needle, resulting in favorable cost-effectiveness.

This study further confirms the favorable tolerability and safety of this novel approach. No significant changes were observed in renal function or urinary protein levels before and after the procedure, and no procedure-related complications occurred.

## Conclusion

In summary, this modified neurolysis demonstrates preliminary efficacy in BP reduction with a favorable safety profile. Its advantages include being contrast-free, preservation of renal artery integrity, procedural simplicity, and cost-effectiveness. These features position it as a promising alternative antihypertensive strategy, particularly for patients who are unsuitable for catheter-based RDN therapy.
